# FSH Promotes Progesterone Synthesis by Enhancing Autophagy to Accelerate Lipid Droplet Degradation in Porcine Granulosa Cells

**DOI:** 10.3389/fcell.2021.626927

**Published:** 2021-02-16

**Authors:** Qiang Liu, Hui Gao, Feng Yang, Hanxue Zhang, Shenming Zeng

**Affiliations:** Laboratory of Animal Embryonic Biotechnology, National Engineering Laboratory for Animal Breeding, Key Laboratory of Animal Genetics, Breeding, and Reproduction of the Ministry of Agriculture, College of Animal Science and Technology, China Agricultural University, Beijing, China

**Keywords:** autophagy, PI3K/JNK/c-Jun, granulosa cell, lipid droplets, progesterone

## Abstract

Little is known about the molecular relationships among follicle stimulating hormone (FSH), lipid droplet (LD) degradation, and autophagy. In this study, we aimed to investigate the pathway by which FSH regulates autophagy and the potential role of autophagy in progesterone production. Our results revealed that FSH stimulated progesterone production in mammalian follicular granulosa cells (GCs) through a non-canonical pathway. In porcine secondary follicles cultured *in vitro*, FSH treatment increased the level of the autophagic marker, LC3-II, as well as increased the number of autophagic vacuoles in GCs. The underlying molecular mechanism and biological functions were then investigated in porcine GCs. Our results demonstrated that FSH could upregulate Beclin1 levels in porcine GCs; however, this effect was blocked by LY294002 (a PI3K/AKT inhibitor) and SP600125 (SAPK/JNK inhibitor). Further research confirmed that the transcriptional factor, c-Jun, was phosphorylated by FSH, then translocated into the nucleus from the cytoplasm and bound to the *BECLIN1* promoter region, and that LY294002, SP600125, or *c-Jun* knockdown prevented the increase in Beclin1 levels induced by FSH. Interestingly, inhibition of autophagy using chloroquine or SP600125 decreased progesterone production in porcine GCs treated with FSH, although the expression of *StAR* and *P450scc* was not disturbed. Moreover, FSH treatment reduced the average number and size of LDs in porcine GCs, but these effects were eliminated by knocking down the key autophagy genes, *ATG5* and *BECLIN1*; in addition, the effect of FSH on promoting progesterone secretion by the cells was also reduced significantly. Based on the above results, we concluded that FSH promoted progesterone production by enhancing autophagy through upregulation of Beclin1 via the PI3K/JNK/c-Jun pathway to accelerate LD degradation in porcine GCs, independent of the classical steroidogenic pathway.

## Introduction

Ovarian follicle development is a dynamic process during which interactions between pituitary gonadotropins and numerous autocrine/paracrine growth factors from the ovary determine whether the follicles grow continuously or undergo atresia (Yuan et al., 2015). Pituitary-secreted follicle stimulating hormone (FSH) is a principal factor that stimulates follicle development, acting from primary to dominant preovulatory follicles (Lee et al., 2001). FSH binds to a G protein-coupled receptor (GPCR), thereby activating several signaling molecules, such as cAMP-dependent protein kinase (PKA), protein kinase B (PKB), p38MAPK (MAPK14), and ERK1/2 (MAPK3/1), and to subsequently promoting the transcription of downstream genes (Wayne et al., 2007), including the steroidogenic acute regulatory protein (StAR), P450 side chain cleavage enzyme (P450scc), inhibin, and activin (Otsuka et al., 2001). Recently, new functions of FSH have been revealed in mammalian follicular development; for example, FSH can promote the survival of porcine granulosa cells (GCs) by decreasing the level of the pro-apoptotic protein, BimEL (Wang et al., 2012), and is involved in autophagy regulation in rat GCs (Choi et al., 2014).

Autophagy is an evolutionarily conserved mechanism that plays an indispensable role in the recycling of intracellular proteins and organelles to maintain cellular homeostasis. It is tightly regulated by a series of autophagy-related gene (Atg) proteins. In particular, Beclin1, the mammalian ortholog of Atg6, plays a central role in autophagy and regulation of membrane trafficking (Kang et al., 2011). Beclin1 can initiate autophagosome formation by binding Vps34 and Vps15, thereby forming the class III PI3K complex to induce autophagy. Previous studies have shown that a number of transcription factors are involved in regulation of *Beclin1* expression, including *p65*, *FoxO1*, *FoxO3*, *c-Jun*, and *E2F1* (Copetti et al., 2009; Li et al., 2009; Wang et al., 2010; Xu et al., 2011; Sanchez et al., 2012). On the contrary, as a Bcl-2-homology (BH)-3 domain only protein, Beclin1 interacts with other apoptotic Bcl-2 family members, which in turn regulates autophagy (Oberstein et al., 2007). Binding of Beclin1 to the anti-apoptotic protein Bcl-2 has been reported to inhibit autophagy (Pattingre et al., 2005). *Beclin1*-null mice exhibit defective in autophagy and early embryonic lethality (Yue et al., 2003); further, targeted deletion of *Beclin1* in the mouse perinatal ovary, resulted in the loss of germ cell populations (Gawriluk et al., 2011). Apart from its classical roles in environmental adaptation, autophagy also participates in the reproductive processes such as fertilization (Tsukamoto et al., 2008), elimination of paternal mitochondria in early embryos (Al Rawi et al., 2012; Sato and Sato, 2012), and accumulation of lipid droplets (LDs) for progesterone synthesis in mouse luteal cells (Gawriluk et al., 2014). Additionally, autophagy modulates foam cell lipolysis to generate free cholesterol (Ouimet et al., 2011), and inhibition of autophagy decreases testosterone production in rat leydig cells (Li et al., 2011; Gao et al., 2018). Studies have shown that intracellular LDs can store triglycerides and cholesterol esters, providing fatty acids or cholesterol for membrane biosynthesis and steroidogenesis (Beller et al., 2010; Brasaemle and Wolins, 2012).

Therefore, progesterone secretion is inseparable from autophagy and LD metabolism, while FSH can promote progesterone secretion. However, little is known about the molecular relationships among FSH, LD degradation, and autophagy. In the present study, the pathway by which FSH regulates autophagy and the potential role of autophagy in progesterone production has been investigated in porcine GCs.

## Materials and Methods

All porcine sample collection procedures were performed in accordance with the ethical principles of animal experimentation approved by Animal Ethics Committee of the China Agricultural University.

### Materials

Unless otherwise specified, all chemicals used in this study were purchased from Sigma-Aldrich (St. Louis, MO, United States). Primary antibodies were purchased from Cell Signaling Technology (Boston, MA, United States). The TFSEARCH program^1^ was used to predict transcription factor -binding sites in the promoters of porcine *BECLIN1*.

### Follicle Isolation and Culture

Porcine ovaries were obtained from the local abattoir and transported to the laboratory in a thermos flask (33–35°C) containing sterile physiological saline within 2–3 h after collection. In the laboratory, the ovaries were washed twice with sterile physiological saline containing 100 IU/mL penicillin and 50 mg/mL streptomycin pre-warmed to 37°C. The ovarian cortex was cut into 500-μm thick sections using the Thomas Stadie-Riggs Tissue slicer (Thomas, 6727C10) and was cross-chopped into 1 mm × 1 mm pieces using the Mcl1-wain Tissue Chopper (Ted Pella, 10180-220). The tissue was then rinsed with DMEM/F12 (Gibco, 11039) supplemented with 1% fetal bovine serum (Hyclone, SH30070.03). Only follicles that contained a clear, centrally located oocyte and healthy brighter GCs were isolated using an 18-gauge needle attached to a disposable 1-mL syringe. Follicular diameter at the beginning of culture was 150–250 μm. The follicles were individually cultured in wells of 96-well plates (Nuclon, EW-01930-10) containing 100 μL of DMEM/F12 supplemented with 3 mg/mL BSA with or without (the control) FSH (Sioux Biochemical, no. 915) in the humidified air with 5% CO_2_ at 37°C, and the medium was changed every 2 days.

### Transmission Electron Microscopy Analysis

After porcine follicles were treated in the presence or absence of 0.01 IU/mL FSH for 24 h, the follicles were prefixed with 2% glutaraldehyde in PBS at 4°C, treated with 1% OsO4 for 3 h at 4°C, and dehydrated in a graded series of ethanol, and then embedded in Epon. Ultrathin sections were mounted on copper support grids in serial order, contrasted with lead citrate and uranyl acetate, and observed using the JEOL JEM-1230EX electron microscope at 80 kV.

### Porcine Granulosa Cell Culture and Treatment

Porcine ovaries were collected at a local abattoir and transported to the laboratory in a thermos flask (33–35°C) containing sterile physiological saline within 2–3 h after collection. In the laboratory, ovaries were washed twice with sterile physiological saline containing 100 IU/mL penicillin and 50 mg/mL streptomycin pre-warmed to 37°C. The GCs were isolated by puncturing the follicles (3–5 mm) with a 25-gauge hypodermic needle and were gently washed thrice with DMEM/F12 containing 1% fetal bovine serum, 100 IU/L penicillin and 100 mg/L streptomycin. GC mass (GC mass) were selected and cultured in DMEM/F12 supplemented with 3 mg/mL BSA in a 35 mm dish (0.5−1 × 10^7^ cells) with or without (control) FSH for the indicated time periods according to the experimental design. For examination of autophagic flux, the LC3 turnover assay was performed. GC mass were cultured in DMEM/F12 medium containing 0.01 IU/mL FSH for 18 h and then chloroquine was added for a further 6 h. For treatment with each specific inhibitor, GC mass were pre-treated with each of these inhibitors for 1 h before the addition of FSH. LY294002 (Beyotime, s1737, 10 mM), SP600125 (Beyotime, s1876, 10 mM), and chloroquine were dissolved in dimethyl sulfoxide and stored at −20°C. The working concentrations of these inhibitors were obtained by dilution (1:1000) with the culture medium.

To obtain monolayer adherent GCs, the follicular fluid was aspirated from the follicle using a 10-mL syringe, washed three times with DMEM containing 10% fetal bovine serum, placed in a 35 mm dish (0.5−1 × 10^7^ cells), and incubated at 37°C. After 12 h of culture, the supernatant was discarded, and the medium was changed. The cells were allowed to grow for 24 h, then passaged and treated. Adherent GCs from porcine ovaries were cultured in DMEM/F12 (Gibco, 11039) serum-free medium with or without (control) oleic acid (OA, 0.5 mM) for 12 h, and then cultured in DMEM/F12 serum-free medium supplemented with FSH or both of FSH and OA for a further 24 h.

### Western Blotting

The cells were lysed in the Laemmli sample buffer (Bio-Rad, 1610737). Equal amounts of protein were separated by SDS-PAGE (12% acrylamide running gel) and transferred to a nitrocellulose membrane (Pierce, 88025). After blocking with 5% non-fat milk in Tris-buffered saline (10 mM Tris, 150 mM NaCl, pH 7.5) containing 0.1% Tween-20 (TBS-T) for 1 h at room temperature, the membrane was incubated overnight with primary antibodies at 4°C. After washing thrice with TBS-T, the membrane was incubated for an additional 1 h with the appropriate secondary antibodies conjugated to horseradish peroxidase at a dilution of 1:3000. The protein bands were visualized using an enhanced chemiluminescence detection system (Applygen Technologies Inc., P1010). Western blot images were processed using the ImageJ software (Wayne Rasband, United States). The primary antibodies used in this experiment were as follows: anti-AKT (Cat# 4685S, RRID:AB_2225340), anti-p-AKT (Cat# 4060S, RRID:AB_2315049), anti-p-JNK (Cat# 4668S, RRID:AB_823588), anti-JNK (Cat# 9252S, RRID:AB_2250373), anti-p-c-Jun (Cat# 3270S, RRID:AB_2129575), anti-β-Actin (Cat# 4970, RRID:AB_2223172), and anti-Atg5 (Cat# 2630S, RRID:AB_2062340) were purchased from Cell Signaling Technology, United States; anti-c-Jun (Cat# Sc-1694, RRID:AB_631263), HRP-labeled goat anti-rabbit IgG (Cat# Sc-2004, RRID:AB_631746) and HRP-labeled goat anti-mouse IgG (Cat# Sc-2005, RRID:AB_631736) were purchased from Santa Cruz, United States; anti-LC3 (Cat# L7543, RRID:AB_796155), anti-Histone3.1 (Cat# ABE154, RRID:AB_2811170), and anti-Beclin-1 (Cat# B6061, RRID:AB_1078276) were purchased from Sigma-Aldrich, United States.

### Cytoplasmic and Nuclear Protein Extraction

Cytoplasmic and nuclear extracts were prepared using the NE-PER nuclear and cytoplasmic extraction kit (Thermo Fisher Scientific, 78833) according to the manufacturer’s instructions. Briefly, the treated cells were washed twice with cold PBS and centrifuged at 500 × *g* for 5 min. The cell pellet was suspended in cytoplasmic extraction reagent I by vortexing. The suspension was incubated on ice for 30 min followed by the addition of cytoplasmic extraction reagent II, vortexed for 5 s, incubated on ice for 90 min and centrifuged for 10 min at 16 000 × *g*. The supernatant fraction (cytoplasmic extract) was transferred to a pre-chilled tube. The insoluble pellet fraction, which contains crude nuclei, was resuspended in nuclear extraction reagent by vortexing for 15 s and incubated on ice for 30 min, then centrifuged for 15 min at 13 000 × *g*. The resulting supernatant, constituting the nuclear extract, was used for the subsequent experiments.

### Reverse Transcription Polymerase Chain Reaction

After culture treatments, total cellular RNA was extracted from GC mass using the TRIzol reagent (Thermo Fisher Scientific, 15596026) and RNA yield was quantified using a NanoDrop 2000 (Thermo Fisher Scientific). cDNA was synthesized using the Superscript First-Strand Synthesis System for RT-PCR (Thermo Fisher Scientific, 18080051) according to the manufacturer’s instructions. The PCR primers used for *beclin1*, *StAR*, *P450scc*, and β-*actin* are shown in Table 1. The gene expression levels were normalized to those of β-actin.

**TABLE 1:** The PCR primer sequences for porcine *beclin1*, *StAR*, *P450scc*, and β*-actin*.

Gene name	sense(5′-3′)	antisense(5′-3′)
*beclin1*	TGGCGGAAAATCTCGAGAAGGTCCA	TGTGCCAAATTGTCCACTGTGCCAA
*StAR*	GGAGAGCCGGCAGGAGAATG	CTTCTGCAGGATCTTGATCTTCTTG
*P450scc*	GTCCCATTTACAGGGAGAAGCTCG	GGCTCCTGACTTCTTCAGCAGG
β*-actin*	ATCGTGCGGGACATAAG	CTCGTTGCCGATGGTGAT

### Chromatin Immunoprecipitation (CHIP) Assay

Porcine GCs were treated with or without (the control) FSH for 24 h. The cells were processed for the CHIP assay according to the manufacturer’s instructions using the EZ-ChIP chromatin immunoprecipitation assay kit (Millipore, 17-371). Briefly, formaldehyde was added directly to the culture medium at a concentration of 1% v/v, and the culture dishes were rotated at 25°C for 10 min. After rotation, glycine was added to the culture medium to a final concentration of 125 mM, and the culture dishes were rotated at room temperature for 5 min. The cells were collected, washed twice with PBS and, lysed in 500 μL of SDS buffer. The lysed cells were sonicated using a Sonics vibra cell, model VCX (Sonics, vibra cell 130) in an ice bath with an amplitude of 30%. The sheared chromatin was harvested and immunoprecipitated using phospho-c-Jun antibody and rabbit normal IgG. Following immunoprecipitation, the beads were washed with chip-wash buffer and the DNA was eluted. PCR amplification was performed using primers spanning the *c-Jun* binding site on the *beclin1* promoter; these are 5′-GCGAACCGACCTACATCCAA-3′ (sense) and 5′-CTTTTTAGGGACCCACCCGC-3′ (antisense).

### Small Interfering RNAs (siRNAs) and Transfection

Control siRNA (siCTR) or negative control siRNA (NC), and nine validated siRNAs against porcine *c-Jun*, *beclin1*, and *Atg5* were designed and synthesized by GenePharma (Shanghai, China). Their sequences are shown in Table 2.

**TABLE 2:** Sequences of siRNA for porcine negative control, *c-Jun*, *beclin1*, and *Atg5*.

siRNA name	sense(5′-3′)	antisense(5′-3′)
siCTR or NC	UUCUCCGAACGUGUCACGUTT	ACGUGACACGUUCGGAGAATT
*sic*-Jun-1^#^	UCCAGUAACGGGCACAUCATT	UGAUGUGCCCGUUACUGGATT
*sic*-Jun-2^#^	GUGCCUACGGCUACAGUAATT	UUACUGUAGCCGUAGGCACTT
*sic*-Jun-3^#^	GCAAAGAUGGAAACGACCUTT	AGGUCGUUUCCAUCUUUGCTT
siBeclin1-1^#^	CCUGGAUCGUGUUACCAUUTT	AAUGGUAACACGAUCCAGGTT
siBeclin1-2^#^	CCAGGAGAGGAGCCAUUUATT	UAAAUGGCUCCUCUCCUGGTT
siBeclin1-3^#^	GCUGGACACUCAGCUCAAUTT	AUUGAGCUGAGUGUCCAGCTT
siAtg5-1^#^	GCUUCGAGAUGUGUGGUUUTT	AAACCACACAUCUCGAAGCTT
siAtg5-2^#^	GCACACCACUGAAAUGGCATT	UGCCAUUUCAGUGGUGUGCTT
siAtg5-3^#^	CCAUCAACCGGAAACUCAUTT	AUGAGUUUCCGGUUGAUGGTT

GC mass were washed thrice with phenol red-free Opti-MEM buffer (Gibco, 31985070), and centrifuged for 5 min at 270 × *g*. The cells were suspended in electroporation buffer, transferred to a 4-mm gene pulsar cuvette (Bio-Rad) with siRNAs and maintained for 5 min at 4°C. The GCs were electroporated at 120 V, with 8 pulses using the BTX ECM 830 electroporator (BTX, United States). Immediately after electroporation, the cells were maintained in a cuvette for 10 min and then suspended in 2 mL of pre-warmed DMEM/F12 containing 3 mg/mL BSA. The cells were transferred to a 35-mm culture dish (Nunc, 174904) and incubated at 37°C for 2 h before further use.

Adherent porcine GCs were transfected with si*Atg5* and si*Beclin1* using the Lipofectamine^TM^ 3000 Transfection Reagent (Thermo Fisher Scientific, L3000008) according to the manufacturer’s instructions. After 6 h of transfection, the cells were treated with FSH for 24 h. Then, the medium was collected for the progesterone assay and the cells were fixed with 4% paraformaldehyde for BODIPY 493/503 (Invitrogen, D3922) staining.

### Fluorescence Microscopy of LDs in Porcine GCs

Adherent porcine GCs treated with siRNA and FSH were fixed with 4% paraformaldehyde for 40 min, and then washed thrice with 1 mL PBS (5 min/wash). LDs were then stained by incubating cells with BODIPY 493/503 for 30 min, and again washed thrice with 1 mL PBS (5 min/wash). The cells were mounted in mounting medium containing Hoechst 33342 stain to highlight the cell nuclei. To detect LDs easily, GCs were pre-treated with oleic acid (OA, 0.5 mM) for 12 h before FSH treatment. BODIPY 493/503 LD staining of adherent GCs after treatment with OA and FSH; Hoechst 33342 staining of the cell nuclei.

Images were acquired with a Leica TCS SP8 fluorescence microscope (Leica Microsystems Inc.) using a 63× objective and 1.4 numerical aperture, subjected to deconvolution using the manufacturer’s software and prepared using the Adobe Photoshop 6.0 software (Adobe Systems Inc.). Quantification was performed in individual frames after deconvolution and thresholding using the ImageJ software (NIH) for a minimum of five cells per slide. LD number and size were quantified with the “analyze particles” function in thresholded single sections with size (pixel 2) settings ranging from 0.1 to 10 and circularity ranging from 0 to 1.

### Progesterone Concentration Assay

The progesterone concentration in the culture medium was analyzed using radioimmunoassay (RIA), which was performed at the Beijing North Institute of Biological Technology, using a progesterone detection kit (Cat. KIP1458) according to the manufacturer’s instructions. Intra- and inter-assay coefficients of variation of all assays were less than 15%. The minimum detectable amount of standard is 0.2 ng/ml.

### Statistical Analysis

Data are presented as the mean ± standard deviation of at least three independent replicates. Data were analyzed by one-way analysis of variance (ANOVA) and the Duncan’s test using the SAS software (SAS Institute, Cary, NC, United States). Differences were considered statistically significant at *P* < 0.05.

## Results

### FSH Increases Autophagy Levels in Porcine Follicular GCs

**FIGURE 1:**
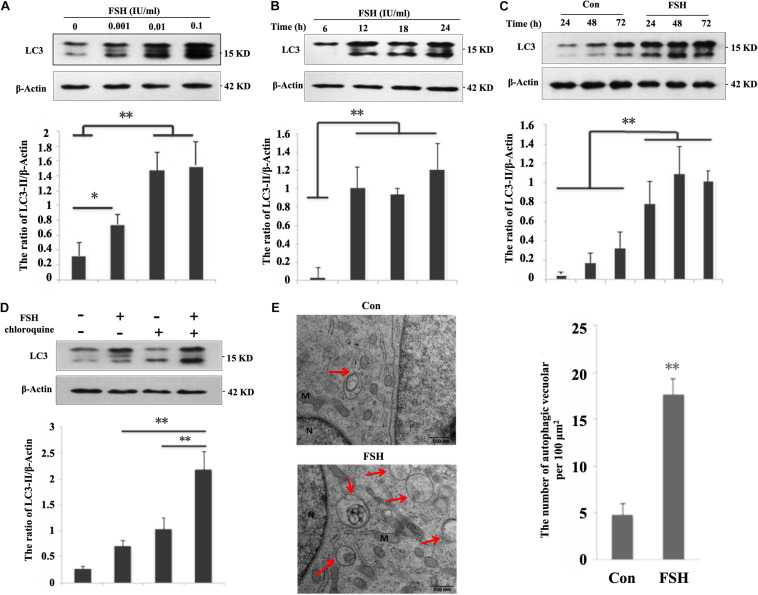
FSH increases autophagy in porcine follicular granulosa cells. **(A)** Porcine follicles were cultured in the DMEM/F12 medium containing 0, 0.001, 0.01, and 0.1 IU/mL FSH for 24 h, LC3-II levels were detected in their lysates. **(B)** Porcine follicles were cultured in DMEM/F12 medium containing 0.01 IU/mL FSH for the indicated time, and LC3-II levels were detected in their lysates. **(C)** Porcine follicles were cultured with 0.01 IU/mL FSH for 24, 48, and 72 h, and LC3-II levels were assessed by immunoblotting their lysates. **(D)** Porcine follicles were cultured in DMEM/F12 medium containing 0.01 IU/mL FSH for 18 h and then chloroquine (10 μM) was added for a further 6 h. LC3-II levels were detected by western blotting. ^∗∗^, *P* < 0.01, and ^∗^, *P* < 0.05 compared to the control group. The value shows the ratio of LC3-II to β-actin and normalized to that of the control. **(E)** Electron microscopic images of autophagic vacuoles in porcine GCs of cultured follicles after treatment with 0.01 IU/mL FSH for 24 h. Autophagosomes are seen (marked by arrows) as rounded vacuolar structures with multiple membranes containing cytoplasmic contents (bars, 500 nm). N: nuclear; M: mitochondria.

To determine whether FSH regulated autophagy at a steady state, we first examined the levels of the microtubule-associated protein 1B light chain 3 (LC3)-II, which is a promising autophagosomal marker for estimating autophagosome quantity and autophagic flux. Greater LC3-II levels were presented by treating porcine follicles with increasing FSH concentrations from 0.001 to 0.1 IU/mL over 24 h (Figure 1A). As treatment with 0.01 IU/mL FSH already yielded a striking increase in LC3-II levels, this concentration was used for subsequent experiments. Thereafter, a time course experiment showed a relatively substantial increase in LC3-II levels during treatment with FSH from 6 to 24 h (Figure 1B). The follicles were then cultured in DMEM/F12 medium supplemented with FSH from 24 to 72 h (Supplementary Figure S1), and LC3-II levels were monitored. As expected, the levels of LC3-II were significantly elevated at 48 h (at 6.3-fold) and at 72 h (3.1-fold) after FSH treatment compared to those in the control (Figure 1C). To clarify whether the increased autophagic vacuoles (AVs) were attributed to the perturbation in autophagic flux by FSH, an LC3-II turnover assay was performed using chloroquine. The results in Figure 1D demonstrated that FSH significantly upregulated LC3-II levels in the presence of chloroquine compared to those with chloroquine or FSH alone. To determine the relationship between FSH and autophagy, electron microscopy analysis was performed to assess the number of AVs in GCs of cultured follicles treated with FSH. The results revealed that the number of AVs per 100 μm^2^ of the porcine follicle GCs cytoplasm was 4.6 ± 1.2 in the control, whereas this number increased to 17 ± 1.6 after FSH stimulation for 24 h (Figure 1E). Taken together, our data suggest that FSH accentuates autophagy in GCs of cultured porcine follicles.

### FSH Upregulates Beclin1 Expression in Granulosa Cells via the PI3K/JNK/c-Jun Pathway

**FIGURE 2:**
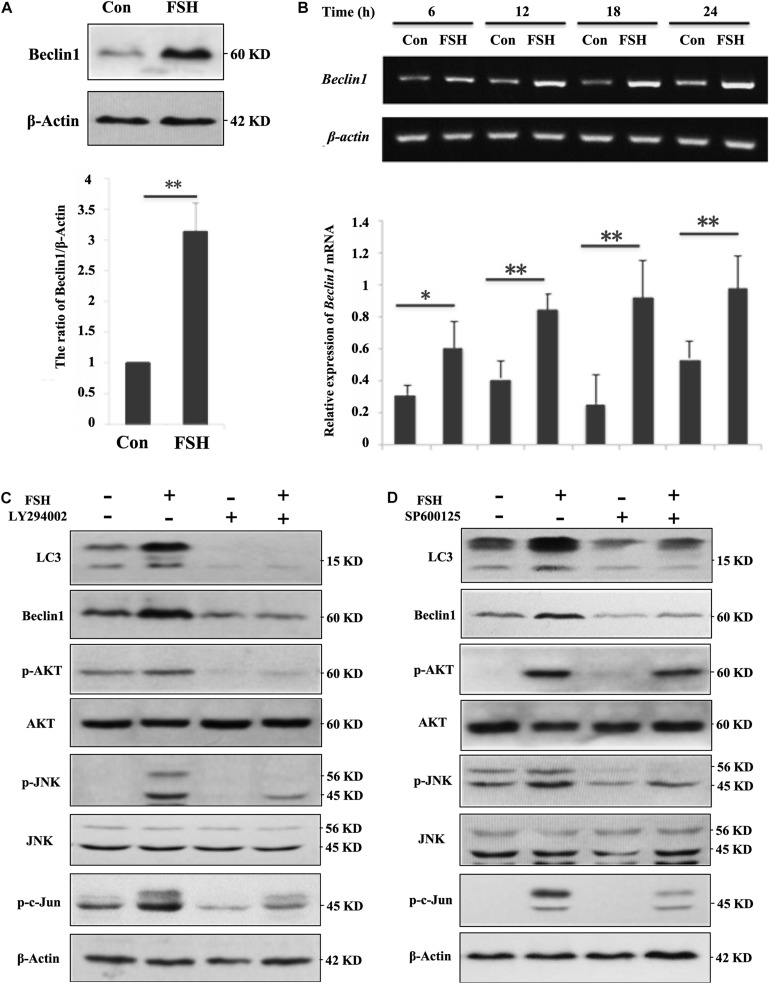
FSH upregulates beclin1 expression in porcine GC mass via the PI3K/JNK/c-Jun pathway. **(A)** GC mass were cultured in DMEM/F12 medium containing 0.01 IU/mL FSH for 24 h, beclin1 and β-actin levels were detected by western blotting of the whole cell lysates. **(B)** GC mass were cultured in DMEM/F12 medium containing 0.01 IU/mL FSH for the indicated hours, and total RNA was collected for cDNA synthesis. Expression of *beclin1* and β*-actin* mRNA was measured by PCR assay. **(C)** GC mass were pre-treated with LY294002 (10 μM, inhibitor of PI3K/AKT signaling pathway) for 1 h before stimulation with 0.01 IU/mL FSH for 24 h, p-AKT, AKT, p-JNK, JNK, p-c-Jun, Beclin1, and β-actin levels were detected in whole cell lysates. **(D)** GC mass were pre-treated with SP600125 (10 μM, inhibitor of SAPK/JNK signaling pathway) for 1 h before stimulation with 0.01 IU/mL FSH for 24 h, p-AKT, AKT, p-JNK, JNK, p-c-Jun, Beclin1, and β-actin levels were detected in the whole cell lysates. ^∗∗^, *P* < 0.01, and ^∗^, *P* < 0.05 compared to the control group.

As LC3-II levels showed similar profiles between porcine follicles (Figure 1D) and GC mass (Supplementary Figure S2) after FSH stimulation, the molecular mechanism by which FSH accentuated autophagy was subsequently investigated in GC mass. Our results demonstrated that FSH notably improved the expression of Beclin1 protein (Figure 2A) and mRNA (Figure 2B). To further explore the signaling pathways involved in the FSH-mediated regulation of Beclin1 expression, LY294002 or SP600125 was employed to block the PI3K/AKT or the SAPK/JNK signaling pathway, respectively; a significant decrease was observed in Beclin1 levels upon LY294002 or SP600125 pre-treatment followed by FSH (Figures 2C,D). This implied that FSH upregulated Beclin1 expression by activating the PI3K/AKT and SAPK/JNK pathways. Furthermore, FSH also increased the phosphorylation of c-Jun (p-c-Jun), which was established as the JNK substrate, whereas the opposite was true for the concomitant treatment with LY294002 or SP600125 (Figures 2C,D). Interestingly, LY294002 not only blocked AKT activation by FSH, but also caused a notable decline in the levels of p-JNK and p-c-Jun. In contrast, pre-treatment of GCs with SP600125 had a negligible effect on p-AKT status. Collectively, these results indicated that activation of AKT and JNK might be required for FSH-mediated Beclin1 expression in GCs.

### C-Jun Is Directly Involved in the Regulation of BECLIN1 Transcription in Response to FSH Treatment

**FIGURE 3:**
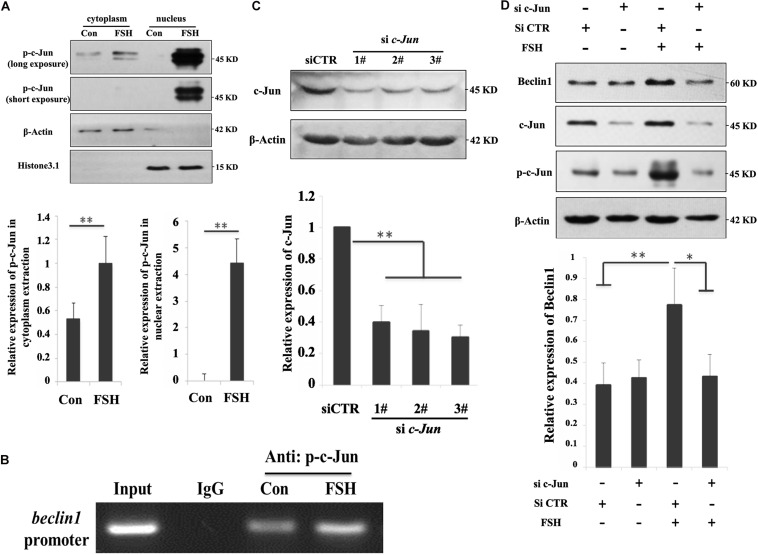
C-Jun was directly involved in *beclin1* transcription in response to FSH treatment. **(A)** GC mass were cultured in DMEM/F12 medium containing 0.01 IU/mL FSH for 24 h, and nuclear and cytoplasmic fractions were prepared from granulosa cells and analyzed by western blotting using the indicated antibodies. Histone 3.1 and β-actin served as the fractionation and loading controls, respectively. ^∗∗^, *P* < 0.01 compared to the control group. **(B)** GC mass were cultured in DMEM/F12 medium containing 0.01 IU/mL FSH for 24 h, the cells were lysed, CHIP assays were performed, chromatin fragments were immunoprecipitated with antibodies against p-c-Jun antibody or normal rabbit IgG, and the *beclin1* promoter was amplified by PCR. The graph of RT-PCR data represents the relative binding by p-c-Jun to the specified DNA-binding elements. **(C)** GC mass were transfected with c-Jun siRNAs (si c-Jun 1-3^#^) and control scramble siRNA (si-CTR) for 24 h and were then harvested and analyzed by immunoblotting with antibodies against c-Jun and actin. **(D)** GC mass were transfected with 3^#^ c-Jun siRNA (si-c-Jun) and control scramble siRNA (si-CTR). At 20 h after transfection, the cells were treated with 0.01 IU/mL FSH for 24 h before harvesting. C-Jun, p-c-Jun, beclin1, and actin were detected in the whole cell lysates. ^∗^, *p* < 0.05; ^∗∗^, *P* < 0.01 compared to the control group.

Given the possible link between c-Jun and Beclin1, we hypothesized that FSH mediated *BECLIN1* expression by enhancing c-Jun binding to its promoter. GC mass were treated with FSH for 24 h to analyze the effect of FSH on the spatial localization of p-c-Jun proteins by western blotting, and most p-c-Jun proteins were congruently detectable in the nucleus (Figure 3A). We next sought to determine p-c-Jun occupancy of the *beclin1* promoter. GC mass chromatins were sheared and immunoprecipitated with p-c-Jun antibody or rabbit IgG prior to RT-PCR analysis using a combination of primers covering the c-Jun presumptive binding sites in the *BECLIN1* promoter. After exposure to FSH, robust recruitment of nuclear p-c-Jun occupancy within the *BECLIN1* promoter was induced but this was significantly less in the control (Figure 3B).

To further substantiate the role of c-Jun in the context of FSH stimulation, an additional approach using siRNA directed against the common sequence of *c-Jun*, was used to knockdown *c-Jun* expression. Among the three different *c-Jun* siRNAs (*sic*-Jun 1–3^#^) assayed, the 3^#^ siRNA effectively reduced the c-Jun protein levels (60%) in the cells compared to those in the NC (Figure 3C). As shown in Figure 3D, a significant reduction in Beclin1 levels was observed upon *c-Jun* knockdown followed by FSH stimulation. Therefore, silencing of *c-Jun* using siRNA blocks FSH-induced Beclin1 expression in porcine GC mass. Taken together, our results indicate that p-c-Jun acts as a direct transcriptional activator by binding to the *BECLIN1* promoter region.

### FSH Participates in Progesterone Production by Regulating Autophagy and LD Metabolism in Porcine GCs

**FIGURE 4:**
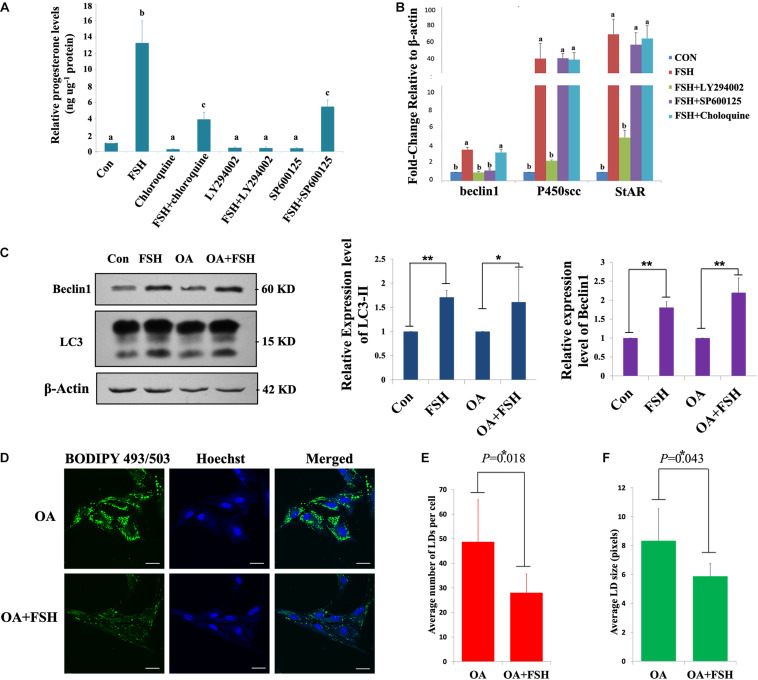
Autophagy participates in progesterone production and lipid droplet metabolism upon FSH treatment in porcine GC mass. **(A)** Specific inhibitors of autophagy (chloroquine; 10 μM), PI3K/AKT (LY294002; 10 μM), and SAPK/JNK (SP600125; 10 μM) were added 1 h before treatment with 0.01 IU/mL FSH for 24 h in GC mass. The cells and culture medium were collected separately. The total proteins were extracted from the cells and quantified using the BCA method. The concentration of progesterone in the culture medium was analyzed using radioimmunoassay. The relative progesterone level in the culture medium was calculated as ng/μg protein. Different superscripts (a, b, c) indicate significant differences (*P* < 0.05) **(B)** Specific inhibitors of autophagy (chloroquine; 10 μM), PI3K/AKT (LY294002; 10 μM), and SAPK/JNK (SP600125; 10 μM) were added 1 h before treatment with 0.01 IU/ml FSH for 24 h in GC mass. Total RNA was extracted from the cells, reverse transcribed to cDNA, and analyzed by real-time PCR with primers against *beclin1*, *StAR*, and *P450scc*. **(C)** Adherent granulosa cells of the porcine ovary were cultured in DMEM/F12 serum-free medium with or without oleic acid (0.5 mM) for 12 h, and then cultured in DMEM/F12 serum-free medium with FSH (0.01 IU/mL) or with FSH and OA (0.5 mM) for 24 h. β-actin, LC3-II, and Beclin 1 levels were detected by western blotting. **(D)** BODIPY 493/503 LD staining of adherent granulosa cells after treatment with OA and FSH; Hoechst 33342 staining of the cell nuclei, Bars: 30 μm. **(E)** The average number of LDs per granulosa cell in the OA and OA + FSH groups. **(F)** The average size of LDs per granulosa cell in the OA and OA + FSH groups. Five visual regions were used for statistics. ^∗^, *P* < 0.05, ^∗∗^, *P* < 0.01.

The role of autophagy in steroidogenesis remains poorly understood. We next sought to functionally connect autophagy with steroid production in GC mass. GCs were pre-treated with chloroquine, LY294002, or SP600125 followed by FSH stimulation to determine their effects on progesterone production. As presented in Figure 4A, we detected a significant increase in progesterone secretion in the GCs after exposure to FSH for 24 h, whereas progesterone levels were notably decreased in the presence of the three chemicals irrespective of FSH stimulation. However, pre-incubation with chloroquine or SP600125 did not affect *StAR* and *P450scc* mRNA levels compared to those in the control (Figure 4B). This suggests that autophagy is involved in progesterone production elicited by FSH and is independent of the classical steroidogenic pathway. To further verify whether FSH regulated lipid metabolism in GCs via the autophagy pathway, the changes in lipid droplets (LDs) were observed in subsequent experiments. To detect LDs easily, GCs were pre-treated with OA for 12 h before FSH treatment. The results demonstrated that OA did not affect the autophagy activities stimulated by FSH (Figure 4C), but the number and size of LDs were decreased significantly after FSH treatment (Figures 4D–F).

### FSH Degrades LDs by Enhancing Autophagy to Promote Progesterone Secretion in Porcine Follicle GCs

**FIGURE 5:**
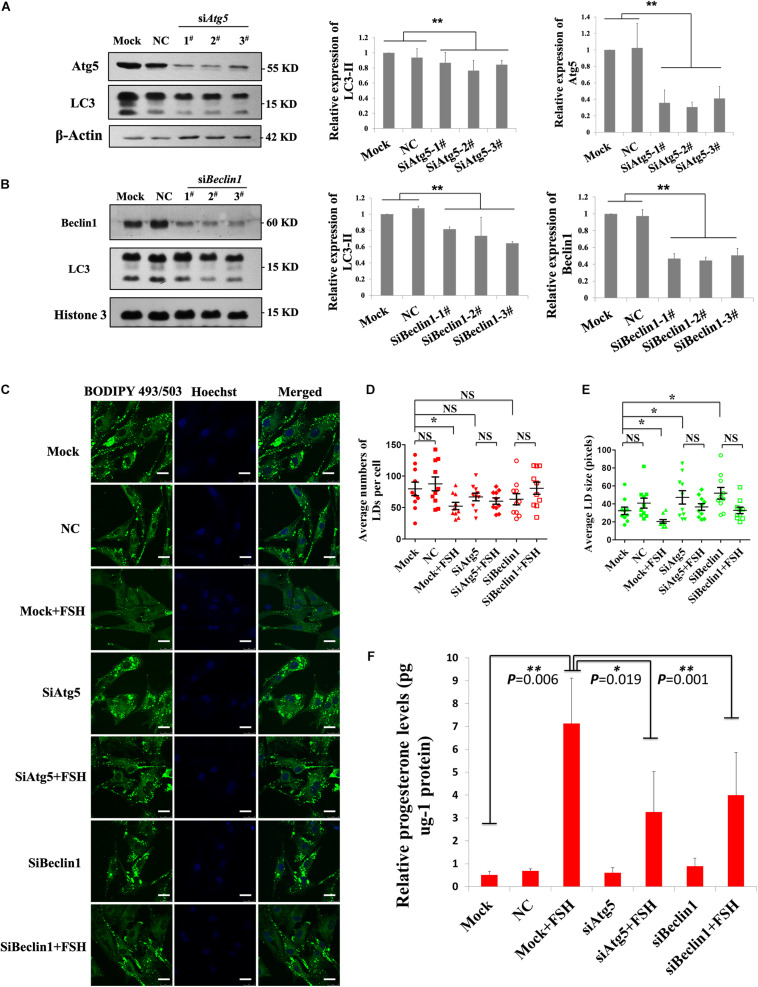
FSH degrades lipid droplets by enhancing autophagy to promote progesterone secretion in porcine follicle GCs. **(A)** Porcine adherent granulosa cells were transfected with Atg5 siRNAs (siAtg5 1-3^#^), NC siRNA, and blank control (Mock) for 24 h and then harvested and analyzed by immunoblotting with antibodies against Atg5, LC3, and actin. **(B)** Porcine adherent granulosa cells were transfected with Beclin1 siRNAs (siBeclin1 1-3^#^), NC siRNA, and blank control (Mock) for 24 h, harvested, and analyzed by immunoblotting with antibodies against Beclin1, LC3, and Histone 3. **(C)** BODIPY 493/503 LD staining of adherent granulosa cells, pre-treated with OA for 12 h, after treatment with NC siRNA, siAtg5 2^#^, and siBeclin1 3^#^, and then treated with FSH for 24 h; Hoechst 33342 staining of the cell nuclei, Bars: 25 μm. **(D)** The average number of LDs per granulosa cell in the Mock, NC, Mock + FSH, siAtg5, siAtg5 + FSH, siBeclin1, and siBeclin1 + FSH groups. **(E)** The average size of lipid droplets per granulosa cell in Mock, NC, Mock + FSH, siAtg5, siAtg5 + FSH, siBeclin1, and siBeclin1 + FSH groups. NC, negative control; NS, no significant. **(F)** The relative progesterone level in the culture medium from mock, NC, mock + FSH, siAtg5, siAtg5 + FSH, siBeclin1, and siBeclin1 + FSH groups was calculated as pg/μg protein. *, *P* < 0.05, **, *P* < 0.01.

To further verify that FSH could enhance the degradation of LDs in GCs by promoting autophagy, the changes in LDs and progesterone secretion in GCs were investigated by knocking down the key autophagy proteins, Atg5 or Beclin1. Among the three different *Atg5* siRNAs (siAtg5 1–3^#^) and three different *BECLIN1* siRNAs (siBeclin1 1-3^#^) assayed, siAtg5 2^#^ and siBeclin1 3^#^ could effectively reduce Atg5 or Beclin1 protein levels in porcine GCs, respectively, compared to those in the control (Figures 5A,B). After knocking down Atg5 or Beclin1 proteins, FSH did not induce the LD changes in the GCs (*P* > 0.05, Figures 5C–E). Compared to those in the control group, the numbers of LDs in the *Atg5* and *Beclin1* knockdown groups were decreased but did not reach statistically significant differences (Figure 5D). However, LD size was significantly higher than that in the control group (*P* < 0.05, Figure 5E). Moreover, progesterone secretion induced by FSH was significantly reduced after knocking down *ATG5* or *BECLIN1* (*P* = 0.019 and *P* = 0.001, respectively, Figure 5F).

## Discussion

FSH plays a vital role in follicle development and steroid production. In this study, we found that FSH activated the PI3K/AKT and SAPK/JNK pathways to stimulate *BECLIN1* expression, leading to upregulated autophagy and LD degradation, which promoted progesterone production independent of steroidogenesis enzymes in porcine GCs. To our knowledge, this is the first evidence demonstrating the relationship among FSH, LDs, and *BECLIN1* expression, and a new pathway to regulate steroid production controlled by FSH was uncovered in mammalian ovary GCs.

FSH binds to its receptor, which exists exclusively on the surface of GCs to stimulate the PI3K/AKT signaling pathway and to activate mTOR (Alam et al., 2004). Given the well-characterized study in which suppression of autophagy by AKT is mediated by mTOR activation (Levine and Kroemer, 2008), we initially hypothesized that FSH inhibited autophagy through the same signaling pathway in GCs. However, our results suggest that FSH induces autophagy. Contrasting observation has been previously reported in mouse GCs where FSH appeared to inhibit autophagy through AKT-mediated activation of mTOR by decreasing LC3-II levels (Choi et al., 2014). Notably, whether FSH plays a direct role in autophagy regulation as an inducer or repressor has not been demonstrated by autophagic flux assay in their experiment (Choi et al., 2014). Similar to our findings, Zhou et al. (2017) reported that FSH treatment could enhance the autophagy level in mouse GCs by upregulating HIF-1a, and FSH-induced autophagy was associated with follicular development. In porcine ovary GCs, our results provided solid data that FSH specifically accentuated autophagy based on LC3-II levels, electron microscopy analysis, and LC3 turnover assay.

Accumulating evidence has demonstrated that mTOR status is neither essential nor sufficient to correlate with the autophagy response in certain cell contexts. For instance, transforming growth factor-β (TGF-β) could activate the AKT-mTOR pathway to inhibit autophagy in fibroblasts (Suzuki et al., 2010; Patel et al., 2012), whereas it increased the expression of *BECLIN1* and *ATG7* to potentiate autophagy in human MDA-MB-231 cells (Kiyono et al., 2009). Moreover, the PI3K-AKT-FoxO3 pathway instead of AKT-mTOR, can induce autophagy by regulating the transcriptional activity of autophagic gene expression in skeletal muscle cells (Mammucari et al., 2008). In human hepatocytes, although hepatitis C virus (HCV) infection enhanced mTOR activation, autophagy was induced through upregulation of *BECLIN1* expression (Shrivastava et al., 2012). Furthermore, mTOR activation could not inhibit autophagy in mouse embryos (Yamamoto et al., 2014).

FSH-mediated accentuation of autophagy may occur through crosstalk with the numbers of distinct signaling pathways and transcriptional control of autophagy-related gene expression. In our results, FSH-mediated activation of PI3K/AKT was found to be necessary for increasing autophagy in GCs. Class I PI3K is a heterodimeric complex comprising a p110 catalytic subunit (α, β, γ, or δ) and a regulatory subunit (p55, p85, or p101) (Li et al., 2013). p110α acts through the AKT-mTOR pathway to inhibit autophagy, whereas p110β co-localizes with the class III PI3K complex, Vps34-Vps15-beclin1-Atg14L, to promote autophagy (Dou et al., 2011). In mouse embryonic fibroblasts, p110β is required for the GPCR signaling and plays an important role in metabolic regulation and glucose homeostasis (Jia et al., 2008). In fact, p110β mediates the action of FSH in mouse Sertoli cells (Guillermet-Guibert et al., 2015). It is reasonable to suggest that FSH may accentuate autophagy through class I PI3K p110β catalytic subunits in porcine GCs. In our experiment, inhibition of the PI3K signaling pathway by LY294002 abolished FSH mediated autophagy in GCs, thereby indicating that FSH regulated autophagy through the PI3K/AKT pathway.

In our study, FSH elicited a comparable increase in the levels of Beclin1 in porcine GCs. It was reported that *BECLIN1* knockout in mouse ovaries caused defects in autophagy and reduced lipid storage in luteal cells, which caused preterm labor (Gawriluk et al., 2014). Several transcription factors regulate *BECLIN1* expression. c-Jun, the JNK downstream substrate, is known to be a transcriptional factor of *BECLIN1*. In Hep3B cells, ceramide activates JNK, inducing the phosphorylation of c-Jun which binds to the specific promoter region to promote *BECLIN1* expression (Li et al., 2009). In addition, FSH upregulated survivin expression through activation of the PI3K/AKT and SAPK/JNK pathways in SKOV-3 cells (Huang et al., 2011). It is possible that FSH can induce *BECLIN1* expression through activation of JNK/c-Jun. In our experiments, both phosphorylation and nuclear localization of c-Jun were markedly increased after FSH treatment, and ChIP-PCR results confirmed that p-c-Jun was bound to the *BECLIN1* promoter region in porcine GCs. Meanwhile, *c-Jun* knockdown by RNAi inhibited the increase in Beclin1 levels induced by FSH, suggesting that c-Jun was directly involved in FSH-mediated *BECLIN1* expression. Interestingly, siRNA could efficiently downregulate c-Jun levels, but failed to inhibit Beclin1 levels in the GCs without FSH treatment, which indicating that *BECLIN1* expression might be regulated by other transcriptional factors in porcine GCs besides c-Jun. According to the *in silico* analysis, the promoter region of the porcine *BECLIN1* gene has several other potential transcriptional factor-binding sites such as for p65 and FoxO1, which is worthy of further study.

Additionally, our results showed that inhibition of autophagosome formation by SP600125 or abolishing autolysosome function by chloroquine could block FSH-mediated progesterone secretion in porcine GCs. Moreover, these effects were independent of steroidogenesis enzymes. This means that FSH promotes steroid synthesis in a new manner in addition to increasing the expressions of *StAR* and *P450scc* in GCs. Interestingly, PI3K inhibition by LY294002 entirely abolished the FSH-mediated progesterone production in porcine GCs. Compared to chloroquine and SP600125, LY294002 pre-treatment significantly inhibited the expression of *StAR* and *P-450scc* mRNA induced by FSH. This indicated that FSH-mediated progesterone production was dependent on both autophagy and steroidogenesis enzymes.

Recently, research on autophagy in lipid metabolism has gradually increased (Liu and Czaja, 2013). In macrophage foam cells, LDs are delivered into lysosomes via autophagy, where lysosomal acid lipase hydrolyzes LDs to generate free cholesterol (Ouimet et al., 2011). Conversely, deletion of *Atg7*, a necessary factor in the autophagy process, causes lipid accumulation in mouse hepatocytes (Singh et al., 2009). Since free cholesterol is the substrate for formation of steroid hormones such as progesterone, autophagy may regulate steroid hormone production. Cholesterol is a key substrate for a series of biological processes and a starting point for steroid hormone synthesis (Miller and Bose, 2011). Studies have shown that autophagy is inextricably linked to LD metabolism and can provide triglycerides and cholesterol to cells by degrading LDs which is called lipophagy (Singh et al., 2009; Zhang et al., 2009). Additionally, progesterone is an indispensable hormone in the reproductive process and plays a role in promoting endometrial development and maintaining pregnancy (Conneely et al., 2002, 2008). However, the specific mechanism by which autophagy participates in progesterone synthesis has not been elucidated. The results from our experiment showed that FSH treatment could significantly reduce the number and size of LDs in porcine ovary GCs. After the inhibition of autophagy by knocking down the key proteins, Atg5 and Beclin1, the effects of FSH treatment on decreasing the amount and size of LDs were eliminated. Simultaneously, the effect of FSH on promoting progesterone secretion was also reduced significantly. However, FSH significantly increased progesterone secretion compared to that in the control group. Therefore, FSH can promote the decomposition of LDs and progesterone secretion in porcine ovary GCs by accentuating autophagy by upregulating Beclin1. Furthermore, consistent with previous studies, the size of LDs was increased significantly after knockdown of Atg5 and Beclin1 in porcine GCs, thereby indicating that inhibition of autophagy promoted LD accumulation (Singh et al., 2009).

Steroidogenesis in GCs is also affected by oocyte-GC communication. Miyoshi et al. (2010) reported that FGF-8 and BMPs, which are growth factors secreted by oocytes, suppressed FSH-induced progesterone production by reducing cAMP in rat GCs. cAMP can induce autophagy through ERK activation in human adipocyte-derived mesenchymal stem cells (Ugland et al., 2011). However, in our study, FSH also activated ERK, which did not correlate with autophagy in GCs (Supplementary Figure S2), suggesting that cAMP might not be involved in FSH-mediated autophagy in porcine GCs. BMPs belong to the TGF-β superfamily, which is closely linked to autophagy regulation. This raises the possibility that oocyte-derived factors modify steroidogenesis by regulating autophagic responses in GCs; future investigation is thus needed to elucidate how oocytes regulate the autophagy of their surrounding cells.

**FIGURE 6:**
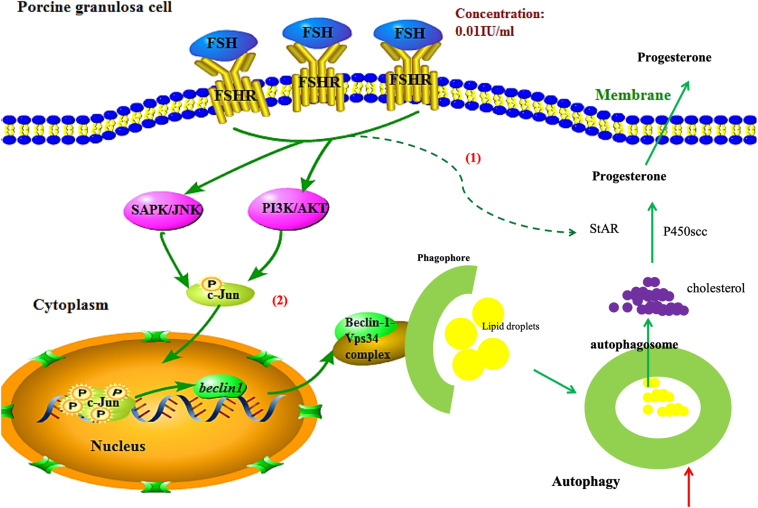
Schematic illustration of autophagy regulation by FSH stimulation in porcine GCs. FSH promotes progesterone secretion by accentuating autophagy through upregulation of Beclin1 via the PI3K/JNK/c-Jun pathway to accelerate LD degradation and increase the expressions of steroidogenesis enzyme genes in porcine GCs.

In summary, FSH promotes progesterone secretion by accentuating autophagy through upregulation of Beclin1 via the PI3K/JNK/c-Jun pathway to accelerate LD degradation and increase the expressions of steroidogenesis enzyme genes in porcine GCs (Figure 6). Our results provide new targets for ovarian hormone therapy and the manipulation of mammalian follicle development.

## Data Availability Statement

The original contributions presented in the study are included in the article/Supplementary Material, further inquiries can be directed to the corresponding author/s.

## Ethics Statement

The animal study was reviewed and approved by Animal Ethics Committee of the China Agricultural University.

## Author Contributions

SZ was involved in conception and design of the project and provided consultation for the analyses. QL and HG collected the samples and performed the experiments. HZ and FY analyzed and interpreted the data. All authors commented on the manuscript and have read and approved the final manuscript.

## Conflict of Interest

The authors declare that the research was conducted in the absence of any commercial or financial relationships that could be construed as a potential conflict of interest.

## Footnotes

1 http://www.cbrc.jp/research/db/TFSEARCH.html

## Supplementary Material

Supplementary Figure 1 Representative photomicrographs of porcine follicles before and after culture.

Supplementary Figure 2 FSH can potentiate autophagy flux in porcine granulosa cell mass (GC mass).
