# Supplementary material for: FSH Promotes Progesterone Synthesis by Enhancing Autophagy to Accelerate Lipid Droplet Degradation in Porcine Granulosa Cells
Source: Front Cell Dev Biol. 2021 Feb 16;9:626927. doi: 10.3389/fcell.2021.626927 (PMC7921800; doi:10.3389/fcell.2021.626927)
Supplement: Supplementary Figure 1 — Representative photomicrographs of porcine follicles before and after culture. [file Table_1.DOCX]

Supplementary Material

## Supplementary Figures


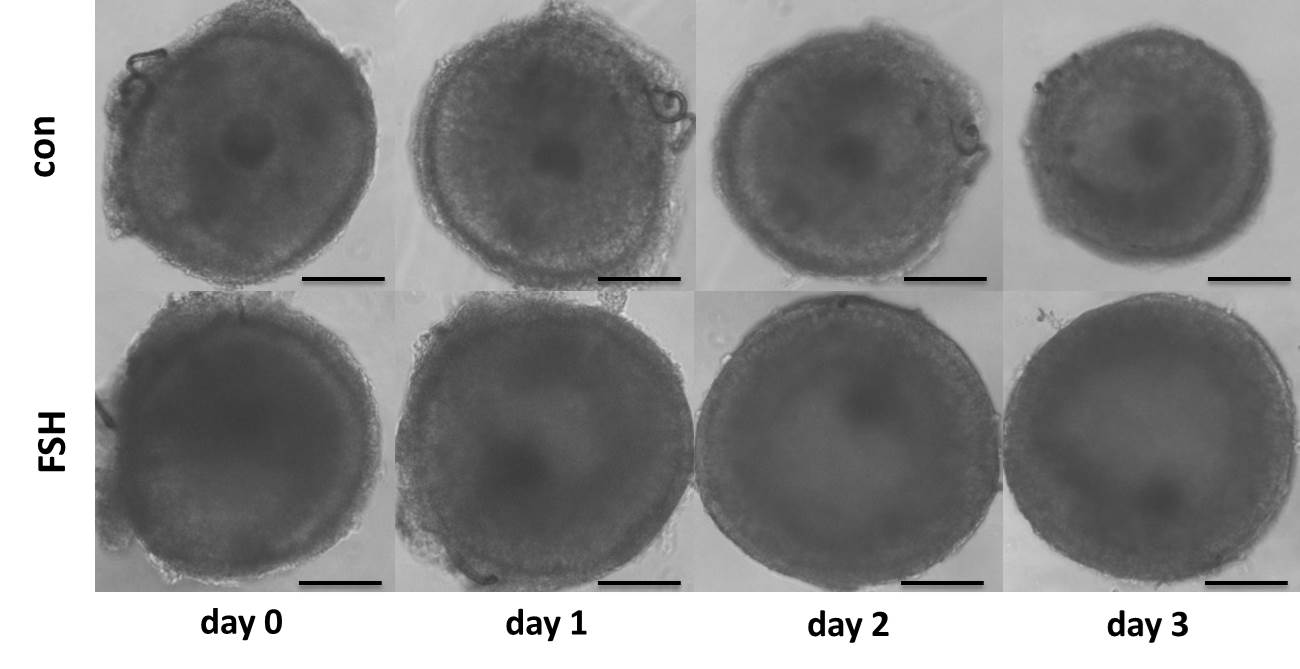


**Figure S1. Representative photomicrographs of porcine follicles before and after culture**

Porcine follicles were cultured in DMEM/F12 medium containing 0.01 IU/mL FSH for 24 h, 48 h, and 72 h (Bars, 100 μm).


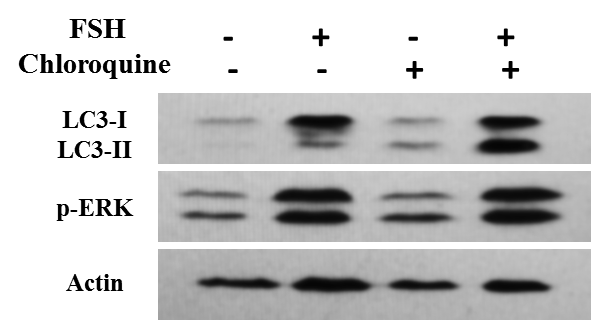


**Figure S2. FSH can potentiate autophagy flux in porcine primary granulosa cells**

Porcine primary granulosa cells were cultured in DMEM/F12 medium containing 0.01 IU/mL FSH for 18 h and then chloroquine (10 μM) was added for a further 6 h. LC3-II levels were detected by western blotting.
